# Translation and Cross-Cultural Adaptation of the Exercise Adherence Rating Scale (EARS) into Danish

**DOI:** 10.1155/2022/4547350

**Published:** 2022-04-12

**Authors:** Julie Sandell Jacobsen, Rasmus Oestergaard Nielsen, Emma Louise Godfrey

**Affiliations:** ^1^Research Centre for Health and Welfare Technology, Programme for Rehabilitation, VIA University College, Aarhus, Denmark; ^2^Research Unit for General Practice, Aarhus, Denmark; ^3^Department of Public Health, Aarhus University, Aarhus, Denmark; ^4^Division of Health and Social Care Research, Faculty of Life Sciences & Medicine, King's College London, London, UK; ^5^Department of Psychology (at Guy's), IoPPN, King's College London, London, UK

## Abstract

The Exercise Adherence Rating Scale (EARS) is a self-administrated questionnaire designed to measure adherence to prescribed home-based exercises in a British population. In a Danish context, no reliable and valid questionnaires are available to measure exercise adherence. This study aimed to translate and cross-culturally adapt the EARS into Danish following international guidelines and to provide insights about construct validity in a Danish population with longstanding hip pain. The EARS was translated and cross-culturally adapted into Danish using a forward-backward method. The understanding and interpretability of the EARS were evaluated with semistructured interviews in 24 patients with longstanding hip pain due to hip dysplasia (22 females; median age 30 (IQR 24-37)). These patients were prescribed home-based exercises. Using Spearman's correlation, construct validity was evaluated by assessing if the Danish version of EARS was correlated with completed exercise sessions and self-reported pain and sport/recreation function. The EARS was translated and cross-culturally adapted into Danish following minor adjustments. The EARS was statistically significantly correlated to completed exercise sessions (*p*=0.005), self-reported pain (*p*=0.005), and sport/recreation function (*p* < 0.03). In patients with longstanding hip pain, the Danish EARS seems suitable to measure adherence to prescribed exercises; however, further evaluation of measurement properties may be needed.

## 1. Introduction

Exercise adherence is the extent to which a person's behavior corresponds with agreed recommendations from a health care provider [1]. Poor exercise adherence negatively compromises the efficacy of exercise interventions since benefits rely on a person's adherence to prescribed interventions [2]. Adherence, therefore, needs to be measured with reliable and valid outcome measures [1, 3]. Without proper measurement of exercise adherence, intervention efficacy can be difficult to determine [1].

In young to middle-aged adults with longstanding hip pain [4], hip-preserving surgery and exercise are the most common treatment modalities [5, 6]. In a research setting, exercise interventions seem inferior in the short term [7, 8], but not in the medium term compared with hip arthroscopy [9]. However, these findings should be interpreted with caution owing to the lack of reporting of exercise adherence. In one study, adherence was not reported [9], in another study, only adherence to supervised sessions was reported [7], and in a third study, only median adherence was reported [8]. In the latter two studies [7, 8], adherence was measured with diaries with no clear predetermined decision rule about the minimum necessary sessions for acceptable adherence. Consequently, it remains uncertain if exercise interventions for hip pain are effective or not. This indicates that the benefit of exercise interventions in young to middle-aged adults with longstanding hip pain, who actually complete the exercise intervention as prescribed, is unknown.

However, exercise adherence is a complex phenomenon, and no reliable and valid outcome measures exist for young to middle-aged Danish adults with longstanding hip pain. The Exercise Adherence Rating Scale (EARS) is a reliable and valid self-reported outcome measure designed to evaluate adherence to prescribed exercise in British patients with chronic low back pain [3]. The EARS has been cross-culturally adapted into other versions [10, 11], and comprehension and face validity of the original British version have also been determined in patients with persistent musculoskeletal pain [12]. However, the EARS has not been cross-culturally adapted for Danish adults with longstanding hip pain.

The present study aimed to translate and cross-culturally adapt the EARS into Danish following international guidelines [13] and to provide insights about construct validity in a Danish population with longstanding hip pain.

## 2. Material and Methods

The EARS consists of six items (section B) that measure adherence. These six items are scored using a 5-item Likert scale from 0 (strongly agree) to 4 (totally disagree). A higher score indicates higher adherence (0–24) (items 1, 4, and 6 are reverse scored). Supporting sections of the EARS have been developed (sections A and C). Section A allows for qualitative information about adherence, while section C includes 10 items related to reasons for nonadherence. Since exercise adherence and not explanations for exercise were the focus of interest, only the EARS items measuring adherence (section B) were translated and adapted.

**Figure 1:**
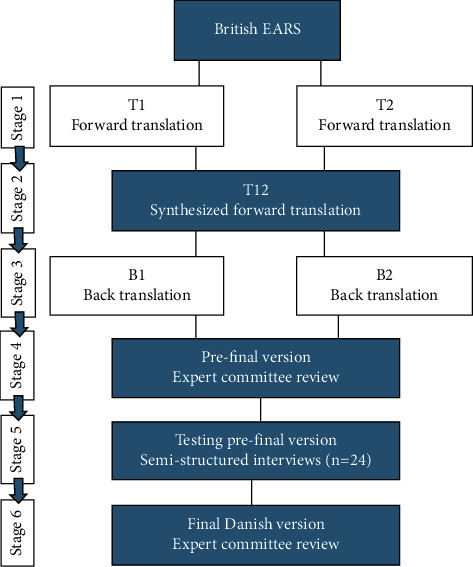
Process of cross-cultural adaptation from the original British version to the Danish Exercise Adherence Rating Scale (EARS).

The EARS was translated and cross-culturally adapted into Danish using an accepted forward-backward method [13]. The first stage was a forward translation. Two translators with Danish as their mother tongue independently translated the EARS from British English into Danish, creating versions T1 and T2 (Figure 1). An accompanied written report was made for each translation, including the rationale for choices and challenging phrases. One translator was a language professional with a Master of Arts in Danish, the uninformed translator (NF). This translator was not aware of the concepts being translated and had no medical or clinical background, offering a translation reflecting the language used in a Danish population. The other translator was a health professional and a researcher with a Ph.D. degree (JSJ). This translator was aware of the concepts being translated, providing equivalency in the translation from a clinical perspective, the informed translator. In stage two, the two translators synthesized the forward translations at a face-to-face meeting. They worked from the original EARS, the forward translations, and written reports and created a synthesized T12 version. All issues arising were documented. During stage three, two back translators blinded to the original version of the EARS, independently translated T12 back into British English, creating versions B1 and B2. The back translators are both English born and have lived in Denmark for more than 20 years. One back translator had no medical or health care background (DT), while the other is specialized in Women's and Men's Health (KL). None of them was aware of the concepts being explored and had no clinical experience with patients with hip dysplasia. In stage four, an expert meeting was held, including a methodologist (RON), a researcher and forward translator with insights into the clinical setting (JSJ), a language professional and forward translator (NF), and one back translator (DT). At the meeting, all versions of the questionnaire (T1, T2, T12, B1, and B2) and the corresponding written reports were consolidated to develop a prefinal version of the questionnaire for field testing. Decisions to achieve equivalence between the source and target version were documented in a report. During this process, the original developer (EG) was in close contact with JSJ and participated in all decisions relevant to the prefinal version. In stage five, the prefinal Danish version of the EARS was tested in 24 adult patients with hip dysplasia (Table 1). The cross-cultural adaptation of the EARS was done as a part of a feasibility study from 2021 (unpublished), where 24 of 30 patients from the feasibility study provided data for the present study. In the feasibility study, patients with hip dysplasia were assessed for eligibility by surgeons from a department of orthopedic surgery at a university hospital in Denmark (January to August 2020). The patients were included if they fulfilled the criteria for inclusion (Table 2). After inclusion, a baseline assessment was completed, and afterward, the patients were instructed to complete home-based exercises a minimum of three times a week for 26 weeks and to report completed exercise sessions in a standardized diary form. After completing the intervention as part of the feasibility study, each of the 24 patients unassisted completed the EARS in a quiet room at the hospital. They were then interviewed about their thoughts on each statement and the chosen answer by vocalizing all thoughts that are normally silent when answering a questionnaire. The interviews were based on a semistructured interview guide developed by author JSJ (Supplemental material, Appendix 1). Afterward, in the final stage six, a written report documented each stage, and versions of the EARS were forwarded to the original developer (EG), and together with the expert committee, she approved the final version.

To offer insights into the interpretation of the EARS in terms of construct validity, we investigated whether the EARS score was correlated with the number of completed exercise sessions and subscales of the Copenhagen Hip and Groin Outcome Score (HAGOS) (pain and sport/recreation) [14]. The patients completed HAGOS at baseline. HAGOS is a reliable, valid, and responsive outcome measure designed to measure pain, symptoms, physical function in daily living (ADL), physical function in sports and recreation (sport/recreation), participation restriction (PA), and quality of life (QOL) in young to middle-aged patients with longstanding hip or groin pain [14]. HAGOS is scored from 0 to 100 points, where 100 is the highest score [14]. We hypothesized a mild to moderate correlation between the EARS and the aforementioned self-reported outcomes, and based on previous studies [10, 11], we hypothesized that the EARS would be positively correlated with completed exercise sessions and positively correlated with self-reported pain and sport/recreation function. The latter two in terms of higher EARS score in a patient with a low level of pain and high functioning.

### 2.1. Analysis

To evaluate the understanding and interpretation of the EARS, 24 patients were interviewed as aforementioned. The interview data for each patient was analyzed separately, generating 144 responses (six items × 24 patients). The data was analyzed in four rounds, and the EARS was prospectively adapted according to issues reported by the patients until no further issues arose.

For the quantitative data, patient characteristics were presented as numbers or as medians with interquartile ranges (IQR) reporting the 25^th^ percentile and the 75^th^ percentile. Spearman's rank correlation coefficient was estimated to evaluate if the EARS score was correlated with the number of completed exercise sessions, baseline HAGOS pain, and baseline HAGOS sport/recreation. Coefficients above 0.7 were classified as strong, those between 0.3 and 0.7 as moderate, and those below 0.30 as weak [15]. STATA 14.2 (StataCorp, College Station, TX, USA) software package was used for the statistical analysis.

### 2.2. Ethics

This study was conducted in accordance with the Declaration of Helsinki, and all patients gave informed consent to participate. The local ethics committee waived the request for approval since noninterventional studies need no formal approval in Denmark (record number: 1-10-72-1-19). The Danish Data Protection Agency authorized the handling of personal data (record number: 1-16-02-301-19).

## 3. Results

During the process of forward and backward translation, adjustments were made to adapt the EARS into Danish (Table 3). In this process, the broader original concept of “exercises/activities” was reduced to exercises since the original term was considered too broad in a Danish context. Moreover, “to which extent” was included to specify that the EARS measures the quantity of exercise adherence, not quality.

In stage five, the prefinal version of the EARS was evaluated in four rounds by the 24 patients as a measure of quality in terms of content validity. Issues were identified in 35% of responses with the majority in round one (42%), reducing to 33% in round two, 17% in round three, and 0% in round four. In round one, the EARS was pretested in six patients, and the majority commented on the introduction, statements 4 and 6. Based on comments from these patients, the EARS was adjusted. In round two, the EARS was pretested in seven patients. This time the comments were centered on statements 3 and 6. Based on comments, the EARS was adjusted a second time. In round three, the EARS was pretested in seven patients. Again, the comments were centered on statements 3 and 6. A fourth and last time, the EARS was further adjusted (round four) and evaluated in four patients. These patients had no misunderstandings, and when interviewed about their thoughts, they interpreted the instruction, items, and responses of the EARS in accordance with the authors' intentions. In addition, the four patients found the text for each item appropriately worded. Finally, the patients reported that the response options matched the question items. Therefore, no further changes were made, and together with the expert committee, the original developer (EG) verified the adaptation process and approved the final Danish version of the EARS (Supplemental material as Appendix 2).

**Figure 2:**
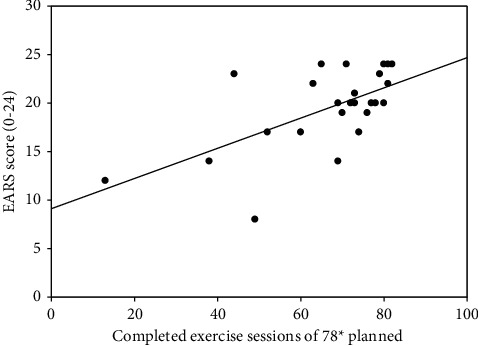
Scatter plot illustrating the correlation between the Exercise Adherence Rating Scale (EARS) and the number of completed exercise sessions (Spearman's *ρ* = 0.56, *p*=0.005). ^*∗*^Patients were instructed to complete a minimum of 78 sessions but were allowed to do more if wanted.

**Figure 3:**
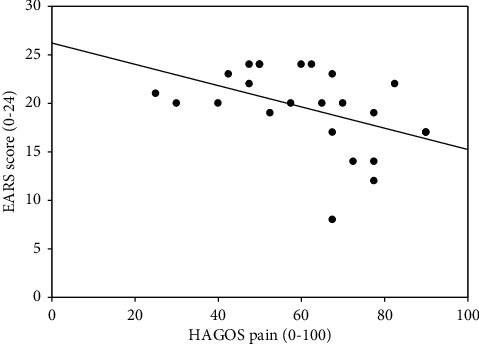
Scatter plot illustrating the correlation between the Exercise Adherence Rating Scale (EARS) and the pain subscale of Copenhagen Hip and Groin Outcome Score (HAGOS) (Spearman's *ρ* = −0.55, *p*=0.005).

**Figure 4:**
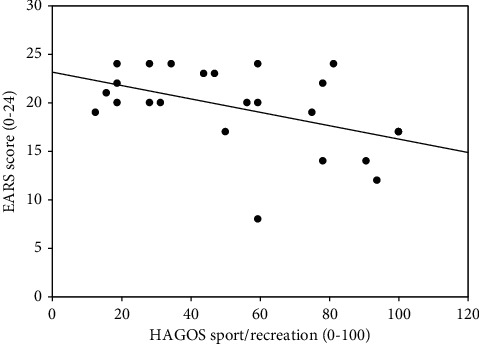
Scatter plot illustrating the correlation between the Exercise Adherence Rating Scale (EARS) and the sport/recreation subscale of Copenhagen Hip and Groin Outcome Score (HAGOS) (Spearman's *ρ* = −0.44, *p*=0.03).

The median EARS score and the median number of completed exercise sessions are reported in Table 1 together with median HAGOS pain and sport/recreation scores, recorded at baseline. The correlation analysis showed a statistically significant correlation between the EARS score and the number of completed exercise sessions, corresponding to a moderate correlation (Table 4) (Figure 2). Similar, statistically significant negative correlations were found between the EARS score and the HAGOS pain (Figure 3) and sport/recreation scores (Figure 4), indicating a moderate reverse relationship between exercise adherence and baseline self-reported pain and sport/recreation function.

## 4. Discussion

The EARS was cross-culturally adapted from British English into Danish applying an internationally accepted forward-backward method [13]. Minor adjustments to the original version of the EARS were made due to linguistic and cultural differences. Despite a lack of in-depth evaluation of measurement properties, we provided insights around construct validity in a small sample of patients, indicating acceptable construct validity. However, further evaluation of measurement properties may be needed.

Other studies have reported cross-cultural adaptation of the EARS using a similar process [10, 11]. Yet, specific linguistic and cultural adjustments in the cross-cultural adaptation process were not reported [10, 11]. Nevertheless, in one study, the cross-cultural adaptation of a Nepali version of the EARS was evaluated in prediabetic adult patients and adult patients with any disease prescribed with home-based exercises [10, 11]. The Nepali version was cross-culturally adapted in 10 patients applying the same forward-backward method [13], as used in our study. The adaptation process revealed no content or language-related issues with no further specifications [10, 11]. The study results also showed a moderate to strong correlation between the EARS score and the 10-item reasons for nonadherence in 18 patients with different diseases [10, 11], and the reliability was acceptable [10, 11]. In another study, the EARS was cross-culturally adapted to Brazilian Portuguese in patients with chronic low back pain by applying the same forward-backward method [10, 11]. The EARS was pretested in 25 patients with chronic low back pain, and measurement properties were investigated in 108 patients with chronic low back pain (CLBP) [10, 11]. Consistent with our evaluation of construct validity, moderate correlations were found between the Brazilian version of the EARS and self-reported pain intensity and disability [10, 11], further supporting our findings. In contrast to our results, the Brazilian EARS was negatively correlated with self-reported pain and sport/recreation function. Our results were surprising since the negative correlation indicates that patients with high levels of pain and low physical function may be more likely to adhere to prescribed exercises compared with patients with low pain levels and high physical function, opposed to the findings in the previous studies [10, 11]. The contrast can be explained by differences in cultural attitudes, perceptions, beliefs, and social interactions. Danish people compared to Brazilian and Nepali populations may have different health care behaviors due to the social-cultural differences [16]. Still, more studies need to confirm this relationship, and if consistent, future studies should investigate what motivates patients with more pain and low function to perform more exercise and whether other social or cultural factors are involved.

The EARS was developed for patients with nonspecific low back pain [3]. However, as aforementioned, the EARS may be applied to any patient who is prescribed home-based exercises by physiotherapists [10, 11]. In a previous study involving the original developer (EG), comprehension and face validity of the EARS were evaluated in 20 patients with persistent musculoskeletal pain [12]. The findings showed that the EARS was comprehensible to the majority of patients with musculoskeletal pain and had a good face validity [12]. The patients reported issues due to confusion between general physical activity and specifically prescribed exercise, issues related to the confusion of the interpretation of specific items with regard to quantity or quality of exercises (i.e., “Is this question saying that I feel confident of the fact that I actually do them, or is it asking that I feel confident when I do them [the exercises]”), and issues related to nonspecification of prescribed exercises (i.e., not received or understood a specific prescription of exercise) [12]. In the light of these comments, the authors recommended refinements to instructions to address these issues [12].

Patients with hip dysplasia have longstanding hip joint-related pain and coexisting muscle-tendon pain [17]. The muscle-tendon pain is anatomically located in the hip [17] and back region [18] and can therefore be considered similar to the patients included in the study by Meade et al. [12] and Beinart et al. [3], which may explain why similar issues arose. In stage four at the expert committee meeting, the main discussion focused on the term “recommended exercises/activities”. The committee agreed on removing “activity” and focusing instead on “recommend exercises”. Similarly, the experts agreed on including “to what extent” to specify a focus and quantity of exercise adherence and not the quality of exercise performance. The last issue, reported in the study by Meade et al. [12], was not commented on in our study. The reason for this may be found in the way the exercises were prescribed to the patients in the feasibility study in terms of supervision of exercise performance and completion of a week-based diary, where all patients received and understood the prescription of exercises.

### 4.1. Strength and Limitations

The EARS was cross-culturally adapted to Danish patients with longstanding hip pain, applying recommended guidelines for cross-cultural adaptation [13]. Furthermore, the original developer (EG) was involved in all processes and approved the final Danish version of the EARS. Moreover, the patients were interviewed under similar conditions (i.e., in a quiet room at the hospital) and by the same interviewer to limit the influence of different conditions. Finally, a standardized semistructured interview guide was used to ensure all relevant aspects were covered. Nevertheless, several limitations to this study do exist. Patients with hip dysplasia are mostly women as reflected in our data. As a result, only two males were involved in the pretesting of the EARS. However, the impact of this is considered minor since the effect of sex on the understanding of items, about how much an individual has been exercising, is likely to be negligible. The participating patients were included in another study. The result of this is a relatively homogenous population, with the potential for the fact that some nuances of the understanding and interpretation of the EARS may have been missed. However, the effect of this is considered minor. During the pretest of the EARS, the questionnaire was adapted using four iterations. As a result, patients completed slightly different versions of the EARS, possibly affecting the responses and the correlation coefficients in the evaluation of construct validity. Nevertheless, the main purpose of this study was to adapt the EARS into Danish based on patients' responses. Therefore, only a preliminary evaluation of construct validity was tested in this study, and extrapolation of our findings to other populations should be made with caution. In spite of this, the Danish EARS seems suitable to measure adherence to prescribed exercises in patients with longstanding hip pain, but further evaluation of its measurement properties may be needed.

### 4.2. Perspective

The Danish EARS has been cross-culturally adapted into Danish in patients with longstanding hip pain. However, further evaluation of measurement properties may be needed, including evaluation of the consistency of the EARS in different populations with musculoskeletal pain and maybe evaluation of performance in any population who are prescribed home-based exercises. Still, the EARS is the first cross-culturally adapted Danish tool to assess adherence to prescribed exercises in patients with longstanding hip pain, and the EARS seems suitable to assess or monitor exercise adherence.

## Figures and Tables

**Table 1:** Characteristics of a patient with hip dysplasia (*n* = 24).

Characteristics	Patients with hip dysplasia
Age, median years (IQR)	30 (24–37)
BMI, median years (IQR)	24 (21–27)
Females/males, no.	22/2
Civil status, no.	
Married, cohabitant, or family status	15
Single or divorced	9
Education, no.	
Primary and lower secondary school	4
Upper secondary education	6
Vocational education	3
Higher education (under- or postgraduate)	11
Duration of pain, no.	
0–2 years	10
2–5 years	7
>5 years	7
Bilateral affection, no.	16
CE angle, median degrees (IQR)	21 (16–22)
AI angle, median degrees (IQR)	12 (10–15)
Tönnis osteoarthritis grade 0/1/2, no.	21/2/1
EARS score, median points (IQR)	20 (17–23)
Completed exercise sessions, median no. (IQR)	73 (62–79)
Baseline HAGOS pain score, median points (IQR)	64 (49–75)
Baseline HAGOS sport/recreation score, median points (IQR)	53 (28–78)

IQR, interquartile range (25–75 percentile); BMI, body mass index); No., number; CE, center-edge; AI, acetabular index; EARS, Exercise Adherence Rating Scale; HAGOS, Copenhagen hip and groin outcome score.

**Table 2:** Inclusion and exclusion criteria for a feasibility study evaluating a six-month exercise and patient education intervention in patients with hip dysplasia.

*Inclusion criteria*

(1). 18–50 years of age
(2). Radiographically verified HD by Wiberg's CE angle of <25 degrees and AI angle >10 degrees
(3). Groin and/or hip pain for a minimum of three months
(4). Not eligible for hip-preserving surgery due to negative impingement test, BMI >25, hip OA, age >45 years, reduced hip range of motion, and/or no wish to undergo surgery

*Exclusion criteria*

(1). Planned arthroplastic hip surgery
(2). BMI >35
(3). Acetabular retroversion defined by crossover sign and posterior wall sign
(4). Legg-Calvé-Perthes disease or epiphysiolysis
(5). Previous pelvic surgery in index limb
(6). Previous pelvic surgery within two years in contralateral limb
(7). Previous surgery due to herniated disc or spondylodesis
(8). Previous arthroplastic surgery in the hip, knee, or ankle
(9). Physical (pregnancy/trauma), neurological, medical, or rheumatic conditions severely affecting the hip function
(10). Inadequacy in written and spoken Danish, mental health issues, or other conditions affecting the ability to follow mandatory stages for participation

HD, hip dysplasia; BMI (Body mass index); OA (osteoarthritis); CE (center edge); AI (acetabular index).

**Table 3:** Adjustments and comments to the Danish version of the Exercise Adherence Rating Scale based on results from expert committee meetings and 24 semistructured interviews.

Item	Original version	Changes	Comments
*Adjustments made during forward and backward translations, stages 1–4* (Figure 1)			
Intro	For each of the following 6 statements, please tick the box which best describes how you do your recommended exercises/activities. When thinking about your answer, please consider any exercises/activities that you have been asked to do as part of your treatment.	For each of the 6 statements, please mark the field, which best describes to which extent you do your recommended exercises. Before answering, please consider all the exercises that you have been asked to do as part of your treatment.	Activity was removed since it was considered too broad in a Danish context, possibly causing confusion between general activity and specifically prescribed exercises.
			“Tick the box” was changed to “mark the field” to make the EARS compatible to different platforms.
			“To which extent” was included to cover quantity and not quality.
			To adapt to Danish, “when thinking about your answer” was changed to “before answering.”
			To adapt to Danish, “any” was changed to “all.”
3 statement	I do less exercise than recommended by my healthcare professional	I do fewer exercises than recommended by the health care professional	Agreement was reached on referring to exercises when possible instead of exercise.
			To adapt to Danish, “my health care professional” was changed to “the health care professional.”
4 statement	I fit my exercises into my regular routine	I fit the performance of my exercises into my daily routine	To adapt to Danish. “regular” was changed to “daily.”
			Choose to use the term “fit the performance” to make sure patients understood that the meaning was the integration of exercise into everyday life.
5 statement	I don't get around to doing my exercises	I do not have the time to do my exercises	To adapt to Danish, “don´t get aground” was changed to “do not have the time to” to specify relation to “time.”
6 statement	I do most, or all, of my exercises	I do most or all my exercises	In Danish, “of” is not necessary and was therefore removed.

*Adjustments made during pretest in 24 patients from the prefinal version to the final version*			
Intro	For each of the 6 statements, please mark the field which best describes to which extent you do your recommended exercises. Before answering, please consider all the exercises that you have been asked to do as part of your treatment.	For each of the 6 statements, please mark the field, which best describes to which extent you do the exercises from your training program. Before answering, please consider all the exercises that you have been asked to do as part of your treatment.	“Your training programme” was added to personalize the introduction.
3 statement	I do fewer exercises than recommended by the health care professional	I do my exercises to a lesser extent than recommended	To specify “quantity,” “fever exercises” were changed to “my exercises to a lesser extent.”
			“Health care professional” was removed since it was unclear who this was.
4 statement	I fit the performance of my exercises into my daily routine	I fit my exercise into my everyday life	To adapt to Danish, “daily routine” was changed to “everyday life.”
			“Performance of my exercises” was changed to “my exercise” since “performance” created several misunderstandings among the patients as well as “my exercises.” The patients understood the performance of exercises and not the integration of exercise into everyday life.
6 statement	I do most, or all, of my exercises	When exercising, I do most, or all, of my exercises	Changes were made since it was unclear whether the statement referred to the number of repetitions, exercises, or sessions per week.

**Table 4:** Correlations between EARS versus the number of completed exercise sessions and baseline self-reported pain and function in sport/recreation (*n* = 24).

Outcomes	EARS	
	*ρ*	*p*-value
Number of completed exercise sessions	0.56	0.005
HAGOS pain	−0.55	0.005
HAGOS sport/recreation	−0.44	0.03

EARS, Exercise Adherence Rating Scale; HAGOS, Copenhagen hip and groin outcome score.

## Data Availability

The data that support the findings of this study are available from the corresponding author upon reasonable request.

## Supplementary Materials

Supplementary Materials Appendix 1: semistructured interview guide used for testing the prefinal version of the EARS. Appendix 2: the Danish version of the EARS and scoring instructions.
