# Supplementary material for: Translation and Cross-Cultural Adaptation of the Exercise Adherence Rating Scale (EARS) into Danish
Source: Transl Sports Med. 2022 Apr 12;2022:4547350. doi: 10.1155/2022/4547350 (PMC11022764; doi:10.1155/2022/4547350)
Supplement: Supplementary Materials — Appendix 1: semistructured interview guide used for testing the prefinal version of the EARS. Appendix 2: the Danish version of the EARS and scoring instructions. [file 4547350.f1.zip › 4547350.f1/Supplemental material_Appendix 2.pdf]

Appendix 2

Exercise Adherence Rating Scale (EARS)

For hvert af de 6 udsagn, marker venligst det felt, der bedst beskriver, **i hvilket omfang** du laver øvelserne i dit træningsprogram. Inden du svarer, bedes du forholde dig til alle øvelser, som du er blevet bedt om at lave som del af din behandling.

1. Jeg laver mine øvelser så ofte som anbefalet

Helt enig				*Helt uenig*
0	*1*	*2*	*3*	*4*
☐	☐	☐	☐	☐

2. Jeg glemmer at lave mine øvelser

Helt enig				*Helt uenig*
0	*1*	*2*	*3*	*4*
☐	☐	☐	☐	☐

3. Jeg laver mine øvelser i mindre grad end anbefalet

Helt enig				*Helt uenig*
0	*1*	*2*	*3*	*4*
☐	☐	☐	☐	☐

4. Jeg tilpasser min træning til min dagligdag

Helt enig				*Helt uenig*
0	*1*	*2*	*3*	*4*
☐	☐	☐	☐	☐

5. Jeg får ikke nået at lave mine øvelser

Helt enig				*Helt uenig*
0	*1*	*2*	*3*	*4*
☐	☐	☐	☐	☐

6. Jeg laver de fleste, eller alle, af mine øvelser, når jeg træner

Helt enig				*Helt uenig*
0	*1*	*2*	*3*	*4*
☐	☐	☐	☐	☐

Scoring af Exercise Adherence Rating Scale (EARS)

EARS scores på en 5-punkt likert skala (0, helt enig til 4, helt uenig). Statement 1, 4 og 6 scores omvendt, hvilket muliggør en score mellem 0 og 24. En højere score indikerer bedre træningsefterlevelse.
