# Supplementary material for: Translation and Cross-Cultural Adaptation of the Exercise Adherence Rating Scale (EARS) into Danish
Source: Transl Sports Med. 2022 Apr 12;2022:4547350. doi: 10.1155/2022/4547350 (PMC11022764; doi:10.1155/2022/4547350)
Supplement: Supplementary Materials — Appendix 1: semistructured interview guide used for testing the prefinal version of the EARS. Appendix 2: the Danish version of the EARS and scoring instructions. [file 4547350.f1.zip › 4547350.f1/Supplementary material_Appendix 1.pdf]

Interview guide for the translation and cross-cultural adaptation of a Danish version of the exercise adherence rating scale (EARS)

Introduction

The purpose of the interviews is to collect information about the understanding of the items and response options for the EARS. The level of interpretation is Steinar Kvale's option of self-understanding. In terms of the theory of knowledge, the approach is hermeneutical.

It is essential to get the respondent to "think out loud" Therefore, the sentence: "please think out loud" is repeated for all items and response options. Nevertheless, it is the interviewer's responsibility to make the conversation flow, and therefore minor adaptations are required in most cases. If the sentence makes no sense to the respondent, the following questions can be used.

1. What are you thinking about when reading the item or sentence?

2. Try to explain in your own words how you understand the item or sentence?

3. You used a considerably long time to answer; what were you thinking about?

Further questions can be added if the respondent has additional comments considered essential to the meaning of an item or response option.

Interview

Presentation
Setting: place the respondent in front of the interviewer and any relatives on a chair behind the respondent. Make sure that the respondent and any relative do not have visual contact.

To the relatives: During the interview, I would kindly ask you to be quiet and please do not make any comment since the respondent's thoughts must be expressed without influence.

To the respondent: Thank you for your time and participation in the interview. I have a few questions about the EARS; please answer the following questions, as it is essential to know if the items are understandable to you. Moreover, we would like to know if the response options are relevant in terms of the exercise that you do.

I would like to record the interview, so please confirm this is alright with you. When reading an item or response option, I would like you to say aloud the first thing you think about. Occasionally, I will also ask questions to fully understand what you are saying.

Introduction

Interviewer: "Can you please read the introduction out loud ".
"Please say what you are thinking about when reading this?"
(If relevant, the interviewer reads the introduction).

"What are you thinking about?"
"What comes to your mind?"
"How do you understand the title?"
"Are there any words you do not understand?"

(Does the patient understand we would like her/him to consider all prescribed exercises?)

Item 1

Interviewer: "Can you please read item one out loud ".
"Please say what you are thinking about when reading this?"
(If relevant, the interviewer reads the item or response options).

"What are you thinking about?"
"What comes to your mind?"
"How do you understand the item?"
"Are there any words you do not understand?"

"Please read the response options."
"How do you understand the response options?"

Item 2

Interviewer: "Can you please read item two out loud".
"Please say what you are thinking about when reading this?"
(If relevant, the interviewer reads the item or response options).

"What are you thinking about?"
"What comes to your mind?"
"How do you understand the item?"
"Are there any words you do not understand?"

"Please read the response options."
"How do you understand the response options?"

Item 3

Interviewer: "Can you please read item three out loud".
"Please say what you are thinking about when reading this?"
(If relevant, the interviewer reads the item or response options).

"What are you thinking about?"
"What comes to your mind?"
"How do you understand the item?"
"Are there any words you do not understand?"

"Please read the response options."
"How do you understand the response options?"

Item 4

Interviewer: "Can you please read item four out loud".
"Please say what you are thinking about when reading this?"
(If relevant, the interviewer reads the item or response options).

"What are you thinking about?"
"What comes to your mind?"
"How do you understand the item?"
"Are there any words you do not understand?"

"Please read the response options."
"How do you understand the response options?"

(Does the patient correctly understand "fit my exercises into my regular routine?")

Item 5

Interviewer: "Can you please read item five out loud".
"Please say what you are thinking about when reading this?"
(If relevant, the interviewer reads the item or response options).

"What are you thinking about?"
"What comes to your mind?"
"How do you understand the item?"
"Are there any words you do not understand?"

"Please read the response options."
"How do you understand the response options?"

Item 6

Interviewer: "Can you please read item six out loud".
"Please say what you are thinking about when reading this?"
(If relevant, the interviewer reads the item or response options).

"What are you thinking about?"
"What comes to your mind?"
"How do you understand the item?"
"Are there any words you do not understand?"

"Please read the response options."
"How do you understand the response options?"

To finish off

Interviewer: " Which item was the hardest to understand and why?"
" Which item was the easiest to understand and why?"
" Do you have anything to add?"
" Thank you for your time and participation."
